# Evaluation of the Efficacy of Mycotoxin Modifiers and Mycotoxin Binders by Using an In Vitro Rumen Model as a First Screening Tool

**DOI:** 10.3390/toxins12060405

**Published:** 2020-06-19

**Authors:** Sandra Debevere, Dian Schatzmayr, Nicole Reisinger, Markus Aleschko, Geert Haesaert, Michael Rychlik, Siska Croubels, Veerle Fievez

**Affiliations:** 1 Department of Pharmacology, Toxicology and Biochemistry, Faculty of Veterinary Medicine, Ghent University, Salisburylaan 133, 9820 Merelbeke, Belgium; sandra.debevere@ugent.be (S.D.); siska.croubels@ugent.be (S.C.); 2 Department of Animal Sciences and Aquatic Ecology, Faculty of Bioscience Engineering, Ghent University, Coupure links 653, 9000 Ghent, Belgium; 3 BIOMIN Research Center, Technopark 1, 3430 Tulln, Austria; dian.schatzmayr@biomin.net (D.S.); nicole.reisinger@biomin.net (N.R.); markus.aleschko@biomin.net (M.A.); 4 Department of Plants and Crops, Faculty of Bioscience Engineering, Ghent University, Valentin Vaerwyckweg 1, 9000 Ghent, Belgium; geert.haesaert@ugent.be; 5 Chair of Analytical Food Chemistry, Technical University of Munich, Maximus-von-Imhof-Forum 2, 85354 Freising, Germany; michael.rychlik@tum.de

**Keywords:** mycotoxins, maize silage, mycotoxin binders, mycotoxin modifiers, efficacy testing

## Abstract

Ruminal microbiota of cattle are not able to detoxify all mycotoxins. In addition, detoxification can be hampered by adverse ruminal conditions (e.g., low ruminal pH). Hence, in the cattle husbandry, mycotoxin binders and modifiers could be used to prevent animal exposure to mycotoxins. In this study, an in vitro rumen model, including feed matrix, was established as first screening tool to test the efficacy of five products claiming to detoxify mycotoxins. The detoxifiers had different modes of action: (a) binding (three products); (b) enzymatic detoxification of zearalenone (ZEN; one product, ZenA); and (c) bacterial transformation of trichothecenes (one product, BBSH 797). For the mycotoxin binders, the binding to the mycotoxins enniatin B (ENN B), roquefortine C (ROQ-C), mycophenolic acid (MPA), deoxynivalenol (DON), nivalenol (NIV), and zearalenone (ZEN) were tested at a dose recommended by the manufacturers. The in vitro model demonstrated that all binders adsorbed ENN B to a certain extent, while only one of the binders also partially adsorbed ROQ-C. The binders did not change the concentrations of the other mycotoxins in the ruminal fluid. The enzyme ZenA detoxified ZEN very quickly and prevented the formation of the more toxic metabolite α-zearalenol (α-ZEL), both at normal (6.8) and low ruminal pH (5.8). The addition of BBSH 797 enhanced detoxification of DON and NIV, both at normal and low ruminal pH. The in vitro rumen model demonstrated that the addition of ZenA seems to be a very promising strategy to prevent estrogenic effects of ZEN contaminated feed, and BBSH 797 is efficient in the detoxification of trichothecenes.

## 1. Introduction

Dairy cattle are often exposed to mycotoxins as high yielders are given a large proportion of maize silage in their ration [1]. Indeed, maize silage is more prone to contamination with multiple mycotoxins compared to grassland products [2,3,4]. In countries with a temperate climate, enniatin B (ENN B), roquefortine C (ROQ-C), mycophenolic acid (MPA), deoxynivalenol (DON), nivalenol (NIV), and zearalenone (ZEN) are often found in maize silage [2,5,6,7]. Previous research has demonstrated that some mycotoxins remain intact in the rumen (e.g., MPA) and that the degradation of mycotoxins is strongly dependent on the rumen conditions [8,9,10]. Hence, for ruminants, detrimental health effects and economic losses in animal production occur, which emphasizes the importance of minimizing mycotoxin exposure.

Numerous strategies have been developed to prevent animal exposure to mycotoxins. As mycotoxins are produced in the field, during post-harvest handling and/or during storage, prevention of mycotoxin production by applying good agricultural practices (GAP) is one of the most important actions to take. Examples of pre-harvest GAP include crop rotation, tillage, use of less susceptible cultivars, application of fungicides, and stem borer control with insecticides [11,12,13]. Examples of harvest and post-harvest GAP are reducing contamination with soil during harvest, reducing humidity level before and during storage, and packing silages tightly to preserve anaerobic conditions [11,12,13]. However, a complete avoidance of mycotoxin contamination of food and feedstuffs is impossible as certain factors that promote mycotoxin production are out of human control, e.g., weather conditions and soil type. Therefore, physical treatments of contaminated grains including density segregation to remove damaged grains, washing and dehulling or reduction of fungal spore contamination by irradiation, or chemical treatments can be applied [11,12,13]. Another commonly used approach to reduce the exposure to mycotoxins in animal feed, is the addition of mycotoxin detoxifying agents (mycotoxin detoxifiers) [11,12,13,14]. Mycotoxin detoxifiers can be divided into two subcategories: adsorbing agents (mycotoxin binders) and biotransforming agents (mycotoxin modifiers). Each subcategory has its own mode of action; binders adsorb mycotoxins in the gastrointestinal tract to enhance the fecal excretion of mycotoxin-binder complexes and prevent mycotoxin absorption, whereas mycotoxin modifiers transform mycotoxins by microorganisms/enzymes into non- or less-toxic metabolites [15].

In contrast to monogastric animals, mycotoxin detoxifiers have not been well investigated in ruminants, except with regards to aflatoxins, given the transfer of their carcinogenic metabolite aflatoxin M1 to the milk. Nonetheless, mycotoxin detoxifiers could also protect ruminants against other mycotoxins since it has been demonstrated that some mycotoxins remain intact in the rumen and ruminal detoxification can be hampered by various factors such as rumen acidosis and microbial activity [8,9,10]. Thus, it is also worth testing the efficacy and safety of mycotoxin detoxifiers in ruminants.

In 2009, the European Food Safety Authority (EFSA) established a functional group of technological feed additives called ‘substances for reduction of the contamination of feed by mycotoxins’ and published guidelines for applicants in 2010 on how to conduct the safety and efficacy assessment of mycotoxin detoxifiers [16]. As a first screening tool, in vitro studies are preferred to reduce the use of laboratory animals [15,17].

In vitro models to study the efficacy of mycotoxin detoxifying agents include static and dynamic models. Static models are suited as a first screening tool as they are cheap and labor efficient. However, they are less representative for the whole gastrointestinal tract and results should be interpreted carefully. Static models are usually based on a buffer system and do not include feed, although feed is always present in the in vivo situation. Dynamic models on the other hand are usually composed of different compartments mimicking the different segments of the gastrointestinal tract [15]. As these models are rather time consuming, require many resources and only allow a limited number of replicates, they are not suitable for first screening experiments. To the authors’ knowledge, no static in vitro model mimicking the rumen metabolism, including both rumen inoculum as well as feed has been reported yet.

In this study, a static in vitro rumen model with the presence of feed was established as first screening tool to test the efficacy of five products claiming to detoxify mycotoxins. The detoxifiers had differently claimed modes of action: (a) binding (three products); (b) enzymatic detoxification of ZEN (one product, ZenA); and (c) bacterial transformation of trichothecenes, such as DON and NIV (one product, BBSH 797). For the mycotoxin binders, the binding to the mycotoxins DON, NIV, ENN B, MPA, ROQ-C, and ZEN were tested.

## 2. Results and Discussion

Four consecutive in vitro experiments were performed:

1. Each mycotoxin binder was mixed into feed. The efficacy of the binders was tested at normal rumen pH (6.8) [18].
2. The enzyme ZenA was added to feed. The efficacy of this mycotoxin biotransforming agent was initially tested at normal rumen pH (6.8).
3. An additional experiment with ZenA was performed to test the activity of the enzyme at different pH levels. Hence, the enzyme was added to the rumen fluid–buffer mixture at two different pH levels just before incubation: low pH (5.8 [18]) and normal pH (6.8).
4. The anaerobic bacterial strain BBSH 797 was added to the rumen fluid–buffer just before incubation as to avoid contact with oxygen. The efficacy of this mycotoxin biotransforming agent was tested at two different pH levels (5.8 and 6.8).

For each experiment, mycotoxin concentrations are expressed relative to the maximal (free) mycotoxin concentration detected during the in vitro rumen simulation study without the addition of the detoxifiers. Hence, the presentation and interpretation of the results is facilitated as mycotoxins might adsorb to feed or other particles in rumen fluid. Indeed, bacteria and yeast cell walls could be involved in adsorbing aflatoxins, DON, ZEN, and fumonisins [19,20,21,22,23,24,25,26,27]. Moreover, we previously demonstrated that ENN B, ROQ-C, and ZEN might be adsorbed to maize silage while MPA was shown to adsorb to rumen fluid particles [28]. During the in vitro incubation, these adsorbed mycotoxins are possibly released, as could be the case when feed particles are degraded. Consequently, mycotoxin concentrations could increase with progressing incubation time as only the free mycotoxin fraction is determined.

### 2.1. Binders in Feed, Normal Rumen pH (6.8)

Just like in our previous study [8], the change in relative mycotoxin concentration compared to the maximal (parent) mycotoxin concentration in rumen fluid is described by means of models which include time, rumen pH, treatment, and interactions between treatment if significant. The final models are given in Supplementary Figures S1–S8 and Tables S1–S6.

On average, over all incubation times, binder 1 reduced ENN B and ROQ-C concentrations by 24% and 11%, respectively, compared to the control treatment, suggesting that the binder adsorbs those mycotoxins to a certain extent. However, addition of binder 1 increased DON concentrations compared to the control treatment, although the small statistical difference of 4.9% (*p* = 0.0124) is irrelevant as this difference falls within the range of analytical precision of the UPLC-MS/MS method (relative standard deviation (RSD) for DON: ±5.2%) [29]. No effect of binder 1 on the MPA, deepoxy-deoxynivalenol (DOM-1), NIV, ZEN, and α-zearalenol (α-ZEL) concentration was observed compared to the control treatment.

According to the provider, binder 1 includes clay minerals and yeast derivatives. The types of clay minerals were not specified by the manufacturer. Clay minerals are mainly effective in binding aflatoxins [15]. Hence, this product could be a good candidate to test binding of aflatoxins in the rumen model. Yeast derivatives are reported to adsorb ZEN by complexation with β-D-glucans in water or buffer solutions [30,31]. The more complex the matrix is (e.g., addition of feed matrix), the higher the chance that the matrix reduces the apparent affinity and capacity of the adsorbents for mycotoxins [15]. Hence, it is possible that the feed particles compete with the adsorbent and hence, the efficacy of the binder decreases [15]. Furthermore, Lauwers et al. demonstrated that the ratio between the concentration of mycotoxins and binder may also influence the binding efficacy [32]. Hence, a too low dose of the product could also have been the reason no effect was seen on the free ZEN concentration.

Binder 2 adsorbed ENN B by 28%, although the difference between the ENN B concentration in the control and binder 2 treatment decreased over time, which could indicate an unstable and reversible adsorption of ENN B. Nivalenol concentrations were statistically higher with binder 2 compared to the control treatment, although the biological relevance of the small difference of 6.7% (*p* = 0.00587) is considered to be negligible as this difference also falls within the range of precision of the UPLC-MS/MS method (RSD for NIV: ±7.8%) [29]. No effect of this binder on the ROQ-C, MPA, DON, DOM-1, ZEN, and α-ZEL concentrations was noted compared to the control treatment.

Binder 2 includes bentonite, leonardite, plant extracts, an epoxidase claimed to be capable to detoxify DON and NIV, and an esterase claimed to be capable to detoxify ZEN. Ramos et al. tested six adsorbents in a simulated intestinal fluid and demonstrated that bentonite is able to adsorb ZEN for only 112.4 µg ZEN/g adsorbent, whereas the best performing binder tested adsorbed 313.7 µg ZEN/g adsorbent, which indicates that bentonite was not the most effective one [33]. In addition, rumen fluid is more complex than simulated intestinal fluid as it contains feed and rumen fluid particles, which could decrease the efficacy of bentonite. Leonardite, an oxidation product of lignite with the highest humic acid content of any natural source, was shown to effectively bind ZEN and reduce toxic ZEN effects in gilts [34]. However, for binder 2, no statistical/relevant effect was seen on the ZEN concentration, despite addition of leonardite and an esterase, or on the NIV and DON concentration, despite addition of an epoxidase, which could be due to the use of a too low dose and/or less optimal conditions for the enzymes’ activity. Another explanation is that ZEN could have a higher affinity for feed particles than for the binder as it has been shown that ZEN could bind to maize silage [28].

ENN B was also adsorbed by binder 3 by 22%. However, as was also observed with binder 2, the difference between the ENN B concentration in the control and binder 3 treatment reduced over time, indicating a reversible binding. Binder 3 supplementation was associated with higher MPA (7.7%, *p* = 0.00656) and NIV (12.8%, *p* < 0.001) concentrations compared to the control treatment. As for the other binders, the small statistical increase seems irrelevant for MPA as this difference falls within the range of precision of the UPLC-MS/MS method (RSD: ±15.5%). Although the difference for NIV exceeds the range of analytical precision of the method, this difference becomes negligible over time due to an interaction between treatment and time [29]. No effect of the binder on the ROQ-C, DON, DOM-1, ZEN, and α-ZEL concentration was seen compared to the control treatment.

Binder 3 is claimed to contain bentonite and sepiolite as active substances. On the one hand, these substances are generally used to bind aflatoxins [15,35]. Hence, this product could be a good candidate to test binding of aflatoxins in the rumen model. On the other hand, Ramos et al. tested six adsorbents in a simulated intestinal fluid and demonstrated that bentonite and sepiolite are also able to adsorb ZEN [33]. In this study, bentonite and sepiolite adsorbed only 112.4 and 74.37 µg ZEN/g adsorbent, whereas the best performing binder tested adsorbed 313.7 µg ZEN/g adsorbent, which indicates that these adsorbents are not the most effective ones. In addition, rumen fluid is more complex than simulated intestinal fluid as it contains feed and rumen fluid particles, which could decrease the efficacy of bentonite and sepiolite. Also, in the case of binder 3, it is possible that a too low dose was used to see an effect on free ZEN concentrations compared to the control treatment.

An overview of the efficacy of the three binders to adsorb mycotoxins can be seen in Table 1. In general, the binders adsorbed a maximum of two of the seven tested mycotoxins. The mycotoxins DON and NIV are hydrophilic, non-ionizable molecules having a rather large epoxide group, which hampers adsorption to plane surfaces. Hence, only very few adsorbing agents are effective DON and NIV binders [15]. Activated carbons are, however, reported to be effective binders for the hydrophilic trichothecenes DON and NIV [15,36]. None of the binders tested contained activated carbons, which could explain why the binders did not lower the free concentrations of the hydrophilic compounds DON, NIV, and DOM-1. However, binder 2 is claimed to contain an epoxidase, which is an enzyme that could have the potential to detoxify DON and NIV [37]. As only one dose of binder 2 was used in this study, the concentration of the enzyme could have been too low, which potentially could be a reason for the lack of effect.

All binders were able to adsorb ENN B by 24% (binder 1), 28% (binder 2), and 22% (binder 3). However, as ENN B is an emerging toxin, little is known about mycotoxin binders that could effectively bind ENN B. Results suggest a stable binding with binder 1, while desorption occurred during incubation with binders 2 and 3. In case of desorption, the toxic compound is released in the gastrointestinal tract and can exert its toxic effect again [38].

The action of binder 1 does not seem to be very specific, as the binder can adsorb both ENN B and ROQ-C, which have different chemical structures. Unspecific adsorption is a major drawback in the use of mycotoxin binders as these products may also adsorb (micro)nutrients and/or veterinary drugs and feed additives such as coccidiostats [39]. Hence, nowadays more and more research is done on targeted modification of the chemical structures of mycotoxins into safe metabolites by using enzymes or microorganisms.

### 2.2. ZenA in Feed, Normal Rumen pH (6.8)

**Figure 1:**
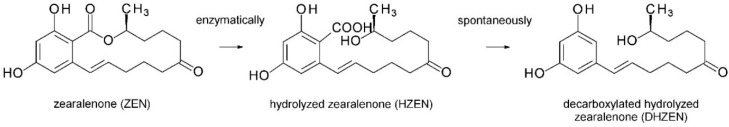
Enzymatical transformation of the estrogenic mycotoxin zearalenone (ZEN) to its hydrolyzed ZEN (HZEN) and decarboxylated HZEN (DHZEN) metabolites by the bacterial enzyme zearalenone hydrolase ZenA. Reprint from Fruhauf et al. [43].

Both, ZEN as well as the metabolite α-ZEL exert estrogenic activity by binding to the estrogen receptor, with α-ZEL being 60 times more potent than its parent toxin ZEN [40]. The detoxifying enzyme used in this study was the bacterial enzyme zearalenone hydrolase ZenA (ZEN*zyme*^®^, BIOMIN Research Center, Tulln, Austria). This enzyme cleaves ZEN to its non-estrogenic hydrolyzed ZEN (HZEN) metabolite, which spontaneously decarboxylates to DHZEN (Figure 1) [41,42].

**Figure 2:**
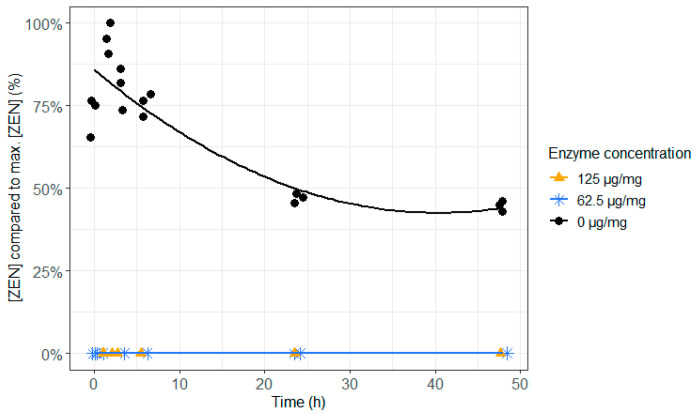
The effect of the zearalenone hydrolase ZenA enzyme, added to the feed, on the zearalenone (ZEN) concentration in the in vitro rumen model. The enzyme was tested at two different concentrations (125 µg/mg feed and 62.5 µg/mg feed) during an incubation period of 48 h. The ZEN concentration is expressed relative to the maximum ZEN concentration detected. As the enzyme degraded ZEN very effectively, its concentration was below the detection limit from the first incubation timepoint and for both enzyme concentrations, resulting in overlapping results of the treatments 62.5 and 125 µg/mg.

**Figure 3:**
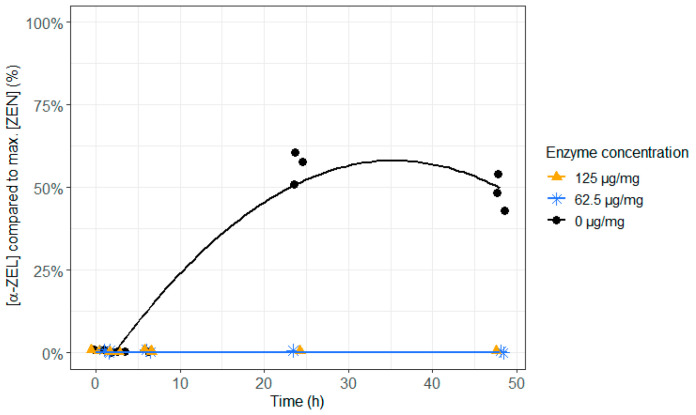
The effect of the zearalenone hydrolase ZenA enzyme, added to the feed, on the molar α-zearalenol (α-ZEL) concentration, formed out of the parent toxin ZEN, in the in vitro rumen model. The enzyme was tested at two different concentrations (125 µg/mg feed and 62.5 µg/mg feed) during an incubation period of 48 h. The molar α-ZEL concentration is expressed relative to the maximum molar ZEN concentration detected. As the enzyme prevented the accumulation of α-ZEL very effectively, its concentration was below the detection limit at all incubation times and for both enzyme concentrations, resulting in overlapping results of the treatments 62.5 and 125 µg/mg.

When the enzyme was not added, almost 50% of ZEN remained intact at the end of the incubation of 48 h and the other 50% of ZEN were entirely transformed to the more toxic α-ZEL. Irrespective of the dose, enzyme supplementation to ZEN contaminated feed resulted in an immediate and complete detoxification of ZEN and prevented the formation of the more toxic metabolite α-ZEL (Figure 2 and Figure 3 and Supplementary Table S7). Hence, adding this enzyme to the feed could prevent reproduction problems caused by ZEN in cattle.

The markedly reduced estrogenic activity of the metabolites HZEN and DHZEN has already been confirmed by Fruhauf et al. in two in vitro models and in an in vivo study with piglets [43]. The MCF-7 cell proliferation assay (0.01–500 nM) and an estrogen-sensitive yeast bioassay (1–10,000 nM) were used as in vitro models. Dependent on the performed test, it was reported that the metabolites HZEN and DHZEN are at least 50–1000 times less estrogenic than ZEN. In the in vivo study, ZEN markedly increased the reproductive tract weight, whereas the ZEN metabolites did not. In addition, uterine mRNA and microRNA expression in female piglets were altered by ZEN but were unaffected by HZEN and DHZEN. These findings support that the enzyme ZenA might be a promising additive to reduce the negative effects of ZEN in dairy cattle as well.

### 2.3. ZenA in Rumen Fluid, Normal (6.8) and Low (5.8) Rumen pH

**Figure 4:**
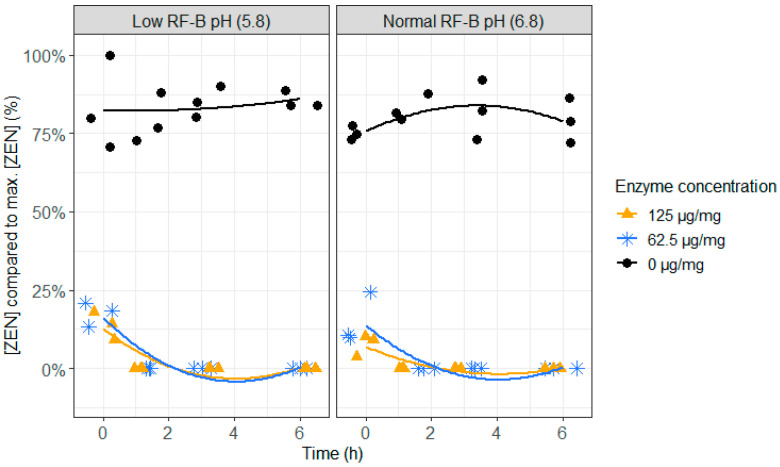
The effect of the zearalenone hydrolase ZenA enzyme, added to the rumen fluid–buffer (RF-B) mixture with low (5.8) or normal (6.8) pH, on the zearalenone (ZEN) concentration in the in vitro rumen model. The enzyme was tested at two different concentrations (125 µg/mg feed and 62.5 µg/mg feed) during an incubation period of 6 h. The ZEN concentration is expressed relative to the maximum ZEN concentration detected. Negative values produced by the model are irrelevant and correspond with 0%.

**Figure 5:**
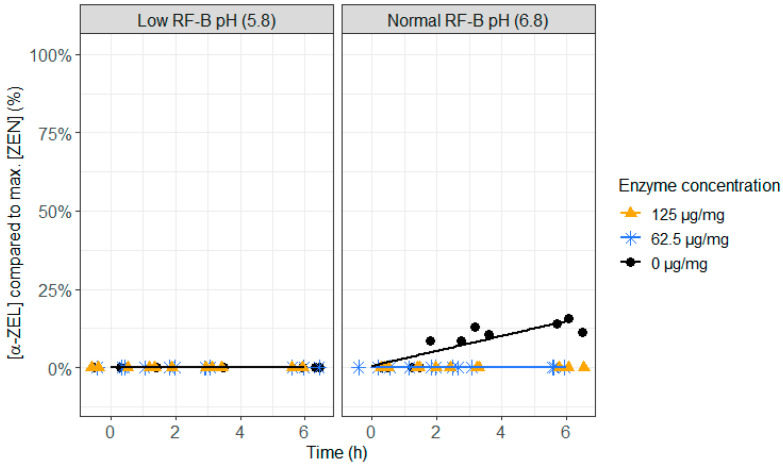
The effect of the zearalenone hydrolase ZenA enzyme, added to the rumen fluid–buffer (RF-B) mixture with low (5.8) or normal (6.8) pH, on the molar α-zearalenol (α-ZEL) concentration, formed out of the parent toxin ZEN, in the in vitro rumen model. The enzyme was tested at two different concentrations (125 µg/mg feed and 62.5 µg/mg feed) during an incubation period of 6 h. The molar α-ZEL concentration is expressed relative to the maximum molar ZEN concentration detected. At the low pH of the RF-B mixture and in all treatments with enzyme addition, α-ZEL accumulation was below the detection limit, resulting in overlapping results.

**Figure 6:**
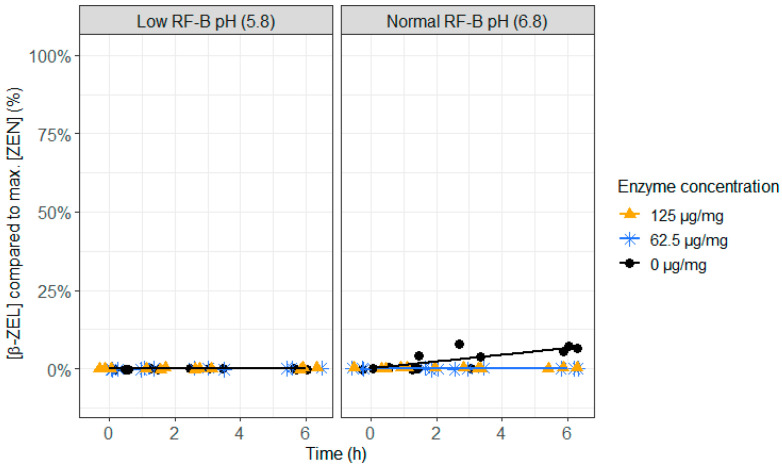
The effect of the zearalenone hydrolase ZenA enzyme, added to the rumen fluid–buffer (RF-B) mixture with low (5.8) or normal (6.8) pH, on the molar β-zearalenol (β-ZEL) concentration, formed out of the parent toxin ZEN, in the in vitro rumen model. The enzyme was tested at two different concentrations (125 µg/mg feed and 62.5 µg/mg feed) during an incubation period of 6 h. The molar β-ZEL concentration is expressed relative to the maximum molar ZEN concentration detected. At the low pH of the RF-B mixture and in all treatments with enzyme addition, β-ZEL accumulation was below the detection limit, resulting in overlapping results.

As the action of ZenA on ZEN should take place in the rumen, it is important to test if ZenA not only detoxifies at normal rumen pH (6.8), but also at low rumen pH (5.8), which can occur during rumen acidosis. Both, at low (62.5 µg/mg feed) and high (125 µg/mg feed) enzyme concentrations, a very fast detoxification of ZEN occurred (Figure 4 and Supplementary Table S8). At the first sampling point (0 h), less than 25% of ZEN was recovered and after 1.5 h of incubation, ZEN was no longer detected. The pH level did not show an effect on the enzyme activity. When no enzyme was added, formation of the more toxic metabolite α-ZEL, and to a lesser extent β-ZEL, occurred at pH 6.8 (Figure 5 and Figure 6 and Supplementary Table S9), which was prevented completely at both enzyme concentrations. This suggests that ZenA is an effective feed additive to degrade ZEN in the rumen of dairy cows and hence to prevent estrogenic effects of ZEN contaminated feed.

### 2.4. BBSH 797 in Rumen Fluid, Normal (6.8) and Low (5.8) Rumen pH

**Figure 7:**
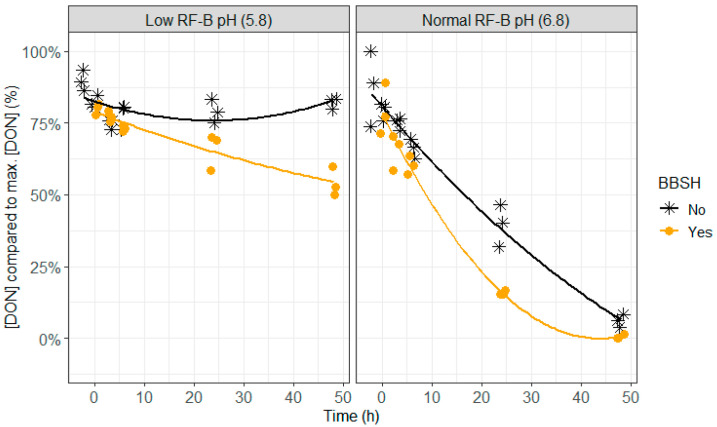
The effect of the mycotoxin detoxifying bacterial strain BBSH 797, added to the rumen fluid–buffer (RF-B) mixture with low (5.8) or normal (6.8) pH, on the deoxynivalenol (DON) concentration in the in vitro rumen model during an incubation period of 48 h. The DON concentration is expressed relative to the maximum DON concentration detected.

**Figure 8:**
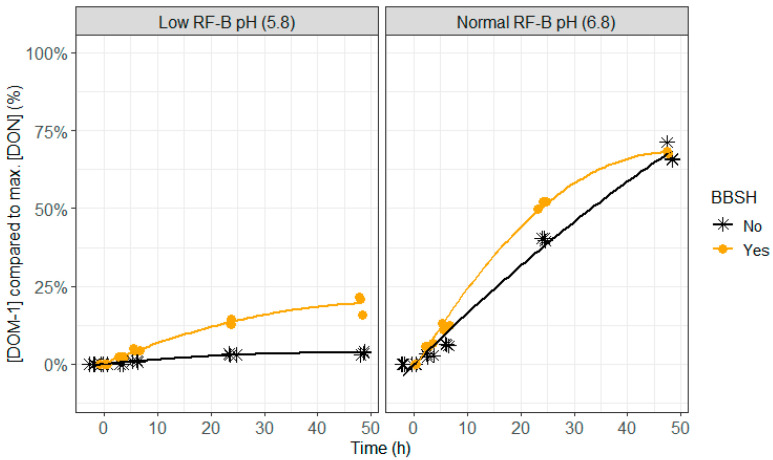
The effect of the mycotoxin detoxifying bacterial strain BBSH 797, added to the rumen fluid–buffer (RF-B) mixture with low (5.8) or normal (6.8) pH, on the molar deepoxy-deoxynivalenol (DOM-1) concentration, formed out of the parent toxin DON, in the in vitro rumen model during an incubation period of 48 h. The molar DOM-1 concentration is expressed relative to the maximum molar DON concentration detected.

**Figure 9:**
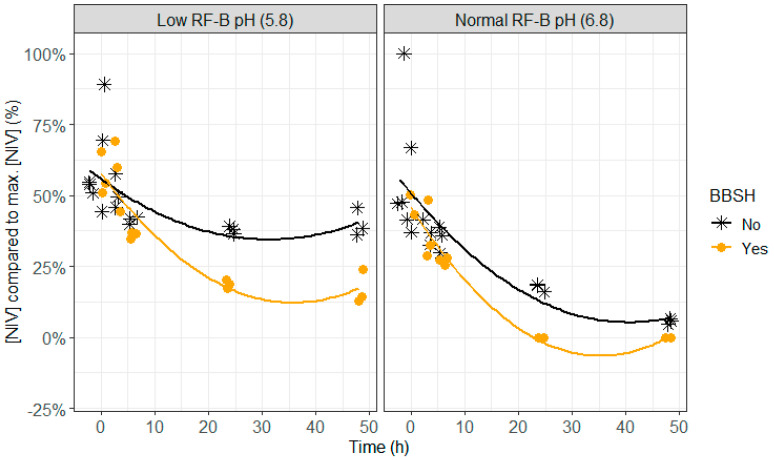
Effect of the mycotoxin detoxifying bacterial strain BBSH 797, added to the rumen fluid–buffer (RF-B) mixture with low (5.8) or normal (6.8) pH, on the nivalenol (NIV) concentration in the in vitro rumen model during an incubation period of 48 h. The NIV concentration is expressed relative to the maximum NIV concentration detected. Negative values produced by the model are irrelevant and correspond with 0%.

Lower DON and NIV concentrations were seen when BBSH 797 was added, which was particularly was obvious after 24 h of incubation at normal pH (6.8) and after 24 and 48 h of incubation at lower pH (5.8; Figure 7, Figure 8 and Figure 9 and Supplementary Tables S10 and S11). At pH 6.8, complete disappearance of DON occurred at 48 h of incubation and of NIV at 24 h of incubation when BBSH 797 was added, in contrast to the control treatment. Also at pH 5.8, DON and NIV had disappeared to a greater extent at the end of the incubation when BBSH 797 was added. This demonstrates the potential efficacy of BBSH 797 to induce a quicker detoxification of trichothecenes in the rumen of cows both at normal and low pH. 

**Figure 10:**
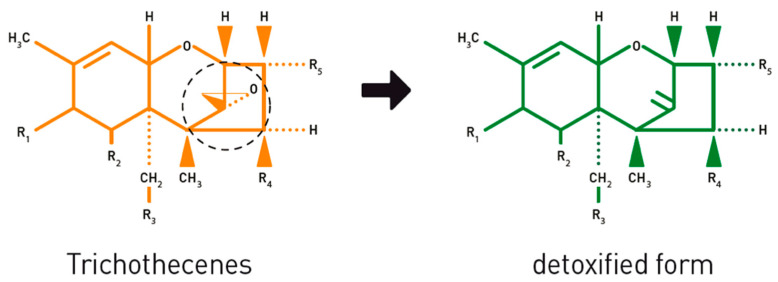
Opening of the toxic 12,13-epoxide ring of trichothecenes, such as DON and NIV, leads to a detoxified form. Reprint from Jenkins [48].

Previous in vitro research has already proven that the bovine rumen microorganism genus *novus* species *novus* BBSH 797 of the *Coriobacteriaceae* family can detoxify trichothecenes by cleavage of the 12,13-epoxide ring (Figure 10) [44]. The safety and efficacy of this ruminal microorganism has been assessed by EFSA for the use in piglets as well as fattening pigs and chickens [45], and has been approved as feed additive for pigs [46] and all avian species by the European Commission [47].

The efficacy of BBSH 797 is not yet reported in a rumen model as ruminants are known to detoxify trichothecenes by their rumen microbiota. In addition, as BBSH 797 is a strain that naturally occurs in the rumen microbiota, it is difficult to obtain significant detoxification data. However, as demonstrated already in a previous in vitro rumen study, the detoxification of trichothecenes can be hampered due to unfavorable rumen conditions [8]. Low pH (5.8) and inoculum from non-lactating cows, which have a lower VFA production compared to inoculum from lactating cows, hampered the detoxification of DON and NIV. In the current study, rumen fluid of dry cows was chosen as the respective microbiota is less active than that of lactating cows and the detoxification of DON and NIV is more challenged. The results of our study are promising, as a clear positive effect of the additive on the detoxification was seen. However, these results should be confirmed with an in vivo trial as in vitro rumen experiments cannot completely mimic the complex nature of the in vivo rumen situation. Nonetheless, in this case, the in vitro situation is less favorable for the strict anaerobic bacterial strain as it is partially exposed to oxygen during the experiment. However, exposure to oxygen was reduced to the minimum as a reducing agent was added to the buffers and the bacterial strain was only added to the incubation flasks after 2 h of incubation when the redox potential should have been stabilized [49].

In conclusion, in vitro results demonstrate that adding BBSH 797 as feed additive is a promising strategy to improve microbial detoxification of trichothecenes in ruminants, especially when detoxification is hampered as during rumen acidosis.

## 3. Conclusions

The described in vitro rumen model, which includes ruminal inoculum as well as a feed matrix, is a suitable first screening tool to investigate the efficacy of mycotoxin detoxifying agents. The model allows easy customization according to the products tested (e.g., anaerobic addition of bacterial strain). This study revealed that all binders adsorbed ENN B to a certain extent while only one of the binders also adsorbed ROQ-C, which has a different chemical structure compared to ENN B. The addition of ZenA is a very promising strategy to detoxify ZEN in contaminated feed and in rumen fluid with normal (6.8) or low (5.8) pH. Also, the addition of BBSH 797 as feed additive is a promising strategy to improve microbial detoxification of DON and NIV in the rumen, both, at normal and low pH.

## 4. Materials and Methods

### 4.1. Rumen Fluid, Maize Silage, Mycotoxins, Chemicals, Reagents, and Mycotoxin Detoxifiers

Rumen fluid was collected from three fistulated Holstein-Friesian cows of 2nd, 4th, and 6th parity, respectively, prior to the morning feeding (EC2015/257; EC of the Institute for Agricultural, Fisheries and Food Research, approved on 10 August 2015). The collected rumen fluid was immediately transferred to the lab in thermos flasks before preparing the buffer–rumen fluid mixture (see Section 4.2). The diet of the cows can be found in Table 2.

Maize silage, as substrate for the incubation, was obtained from Agrivet (Melle, Belgium) in May 2012, and was lyophilized and stored at ambient temperature as standard incubation substrate. A validated multi-mycotoxin LC-MS/MS method was used to determine the mycotoxin concentrations in the lyophilized maize silage sample. This was performed at the Centre of Excellence in Mycotoxicology and Public Health, Department of Bioanalysis at Ghent University (Belgium). Only traces of ROQ-C (12 µg/kg), NIV (201 µg/kg), DON (593 µg/kg), and ZEN (68 µg/kg) were detected. The concentration of ENN B was below the cut-off value of 80 µg/kg.

The analytical standards of ROQ-C, MPA, DON, NIV, and ZEN were purchased from Fermentek (Jerusalem, Israel). The standards of ENN B, DOM-1, α-ZEL, β-ZEL, α-ZAL, β-ZAL, and ZAN were purchased from Sigma–Aldrich (Overijse, Belgium). The internal standards (IS) ^13^C_22_-ROQ-C, ^13^C_17_-MPA, and ^13^C_18_-ZEN were purchased from Food Risk Management (Oostvoorne, The Netherlands). The IS ^13^C_15_-DON was purchased from Sigma–Aldrich. The IS ^15^N_3_-ENN B was synthesized as reported earlier [50].

Methanol (MeOH), acetonitrile (ACN), and water (H_2_O) were purchased from Biosolve (Valkenswaard, The Netherlands) and were of ULC/MS grade. Hydrochloric acid (HCl) 37%, acetic acid (AA), ethanol, and ethyl acetate (EtAc) were obtained from Merck Millipore (Overijse, Belgium) and were of analytical grade. Ammonium hydrogen carbonate (NH_4_HCO_3_) and potassium dihydrogenphosphate (KH_2_PO_4_) were purchased from VWR (Leuven, België), while the phosphate-buffered saline (PBS) powder packs were from Thermo Fisher Scientific (Merelbeke, Belgium). Disodium hydrogen phosphate dodecahydrate (Na_2_HPO_4_·12H_2_O) and magnesium chloride hexahydrate (MgCl_2_·6H_2_O) were from Carl Roth (Vienna, Austria). Sodium hydrogen carbonate (NaHCO_3_), MES (2-(N-morpholino)ethanesulfonic acid, 4-morpholineethanesulfonic acid), and L-cysteine hydrochloride monohydrate were purchased from Sigma–Aldrich (Overijse, Belgium). Carbon dioxide (CO_2_) was purchased from Air Liquide (Aalter, Belgium).

Three mycotoxin binders were tested. Binder 1 was a mixture of clay minerals and yeast derivatives. Binder 2 contained bentonite, leonardite, plant extracts, epoxidase, and esterase. Binder 3 was a mixture of several clay minerals (bentonite and sepiolite). The ZEN detoxifying enzyme zearalenone hydrolase ZenA (ZEN*zyme*^®^) was provided by BIOMIN as well as the BBSH 797 bacterial strain to detoxify trichothecenes such as DON and NIV.

### 4.2. Standard Solutions and Rumen Fluid–Buffer Mixtures

Stock solutions of 1 mg/mL in ACN were prepared for ENN B, ROQ-C, MPA, DON, NIV, and ZEN, and of 100 μg/mL in ACN for α-ZEL, β-ZEL, α-ZAL, β-ZAL, and ZAN. DOM-1 was in solution upon purchase (50 μg/mL ACN). An IS-stock solution of 5 μg/mL in ACN was prepared for ^15^N_3_-ENN B. All other IS were in solution upon purchase, i.e., ^13^C_15_-DON: 25.3 μg/mL in ACN; ^13^C_17_-MPA: 25.4 μg/mL in ACN; ^13^C_22_-ROQ-C: 25 μg/mL in ACN; and ^13^C_18_-ZEN: 25.4 μg/mL in ACN. The standard and IS stock solutions were used to prepare appropriate working solutions for the spiking experiments (see Section 4.3), and matrix-matched calibrator and quality control (QC) samples (see Section 4.4). All stock and working solutions were stored at ≤−15 °C.

The buffer used for the in vitro rumen simulations performed only with normal rumen pH (mentioned under Section 4.3.1 and Section 4.3.2) and for dissolving the BBSH 797 detoxifier contained the following salts per liter: 1.55 g KH_2_PO_4_, 3.58 g Na_2_HPO_4_·12H_2_O, 0.124 g MgCl_2_·6H_2_O, 8.74 g NaHCO_3_, and 1.00 g NH_4_HCO_3_. The buffer was saturated with CO_2_ overnight and kept at 39 °C.

The buffer used for the in vitro rumen simulations performed at low and normal pH (mentioned under Section 4.3.3 and Section 4.3.4) contained the following compounds per liter: 54.31 g MES, 0.124 g MgCl_2_·6H_2_O, 8.74 g NaHCO_3_, and 1.00 g NH_4_HCO_3_. The buffer was also saturated with CO_2_ overnight and kept at 39 °C. Before adding to the rumen fluid, 500 mg of reducing agent L-cysteine hydrochloride monohydrate per liter buffer was added and pH was adjusted with 6 M of HCl to 6.8 or 5.8.

For all rumen fluid–buffer mixtures, fresh rumen fluid from different cows was sieved using a sieve with mesh width of 1 mm, mixed together, and then added to the buffers. The rumen fluid/buffer ratio was 263.2 mL/1000 mL.

### 4.3. In Vitro Rumen Simulation Experiments

#### 4.3.1. Binder in Feed, Normal Rumen pH

Each binder was mixed at a ratio of 3 g per kg maize silage, according to the recommended dose indicated by the manufacturer, with a Turbula shaker mixer (Eskens, Mechelen, Belgium). A spiking solution of mycotoxins was prepared and contained 2.5 µg/mL ENN B, 5 µg/mL ROQ-C, 15 µg/mL MPA, 30 µg/mL DON, 150 µg/mL NIV, and 7.5 µg/mL ZEN, dissolved in ethanol/H_2_O (50/50, v/v). Twenty µL of the spiking solution was added to 50 mg of maize silage with mycotoxin binder in an incubation flask of 25 mL, thus the maize silage contained 1 mg/kg ENN B, 2 mg/kg ROQ-C, 6 mg/kg MPA, 12 mg/kg DON, 60 mg/kg NIV, and 3 mg/kg ZEN. For DON and ZEN, the concentrations were based on the maximum guidance levels in maize by-products published by the Commission of the European Communities [51]. As no maximum guidance levels are available for the other mycotoxins, the concentrations were based on worst case contamination levels in maize silage found in Belgium and the Netherlands [2,5,6,7,52]. After adding 200 µL of distilled water, the incubation flasks were flushed with CO_2_ and 4.8 mL of fresh rumen fluid–buffer mixture was added. The samples were incubated in triplicate at 39 °C in a shaking incubator (Edmund Bühler TH30; Edmund Bühler GmbH, Hechingen, Germany). After 1.5 h, 3 h, 6 h, 24 h, and 48 h, 1 mL of rumen fluid sample was taken to determine the mycotoxin concentrations.

#### 4.3.2. ZenA in Feed, Normal Rumen pH

A spiking ZEN solution was prepared at 7.5 µg/mL ZEN, dissolved in ethanol/H_2_O (50/50, v/v). Twenty µL of the spiking solution was added to 50 mg of maize silage in an incubation flask of 25 mL, to obtain a concentration of 3 mg/kg ZEN. Just before starting the incubation, the zearalenone hydrolase ZenA enzyme was dissolved in demineralized water at two different concentrations, i.e., 15.625 mg/mL (low concentration or 62.5 µg/mg feed) and 31.25 mg/mL (high concentration or 125 µg/mg feed). To each incubation flask, 200 µL of demineralized water (control), or enzyme solution at low concentration or at high concentration was added. The incubation flasks were flushed with CO_2_ and 4.8 mL of fresh rumen fluid–buffer mixture was added. The samples were incubated in triplicate at 39 °C in a shaking incubator. After 1.5 h, 3 h, 6 h, 24 h, and 48 h, 250 µL of the rumen fluid sample was taken to determine the mycotoxin concentrations.

#### 4.3.3. ZenA in Rumen Fluid, Normal and Low pH

This experiment was performed as described in Section 4.3.2 with some small modifications. After adding maize silage and the mycotoxin solution to the incubation flasks, the flasks were flushed with CO_2_ and 4.8 mL of fresh rumen fluid–buffer mixture at pH 5.8 or pH 6.8 was added. To each incubation flask, 200 µL of demineralized water (control), or enzyme solution at low or high concentration was added just before incubation. The incubation lasted only for 6 h with sample collection at 1.5 h, 3 h, and 6 h.

#### 4.3.4. BBSH 797 in Rumen Fluid, Normal and Low pH

A mycotoxin spiking solution was prepared at 30 µg/mL DON and 150 µg/mL NIV, dissolved in ethanol/H_2_O (50/50, v/v). Twenty µL of the spiking solution was added to 50 mg of maize silage in an incubation flask of 25 mL, to obtain a concentration of 12 mg/kg DON and 60 mg/kg NIV. After adding 200 µL of distilled water, the incubation flasks were CO_2_ flushed and 4.8 mL of fresh rumen fluid–buffer mixture was added. The samples were incubated in triplicate at 39 °C in a shaking incubator. After 2 h of incubation, the BBSH 797 detoxifier was dissolved in the CO_2_ flushed phosphate bicarbonate buffer (0.1 g per mL buffer) and 200 µL of the detoxifier (treatment) or buffer without detoxifier (control) was added to the flasks. After another 3 h, 6 h, 24 h, and 48 h of incubation, 250 µL of the rumen fluid sample was taken to determine the mycotoxin concentrations.

### 4.4. Calibrator and Quality Control (QC) Samples Used for Mycotoxin Analysis

Matrix-matched calibration curves were prepared for each combination of rumen fluid–buffer mixture. The appropriate combined working solution was added to 250 µL of rumen fluid–buffer mixture to obtain a calibration range of 0.05–15 ng/mL of ENN B, 0.10–30 ng/mL of ROQ-C, 0.3–90 ng/mL of MPA, 0.6–180 ng/mL of DON and DOM-1, 30–900 ng/mL of NIV, and 0.15–45 ng/mL of ZEN, α-ZEL, β-ZEL, α-ZAL, β-ZAL, and ZAN. Quality control samples were prepared at low (10% of the initial concentration in spiked samples) and high (initial concentration in spiked samples) concentration levels.

### 4.5. Rumen Fluid Sample Extraction and LC-MS/MS Mycotoxin Analysis

Samples from the in vitro experiment with the binders were first centrifuged (3724× *g*, 5 min, 4 °C) to precipitate the binder and mycotoxin-binder complexes. A subsample of 250 µL was taken to extract the mycotoxins. The procedure as described in Debevere et al. [29] was applied to extract ENN B, ROQ-C, MPA, DON, DOM-1, NIV, ZEN, and possible metabolites, with some small modifications. In brief, 25 µL of the IS-mix working solution (10 ng/mL for ^15^N_3_-ENN B and ^13^C_22_-ROQ-C, 100 ng/mL for ^13^C_15_-DON, ^13^C_17_-MPA, and ^13^C_18_-ZEN), 250 µL of PBS, and 1.5 mL of EtAc were added to the 250 µL rumen fluid sample. The samples were vortexed mixed and extracted on an overhead shaker (Trayster digital, IKA, Staufen, Germany) for 15 min. After centrifugation (3724× *g*, 5 min, 4 °C), the upper organic phase was collected and evaporated to dryness under a gentle stream of nitrogen at ~50 °C. The dry residue was redissolved in 200 µL of H_2_O/ACN (85/15, v/v), vortex mixed, filtrated using a 0.20 µm Millex^®^-LG PTFE filter (Merck, Overijse, Belgium), and collected in an autosampler vial.

Samples were injected on an UPLC system consisting of an Acquity H-Class Quaternary Solvent Manager with temperature-controlled tray and column oven from Waters (Zellik, Belgium). Chromatographic separation was achieved on an Acquity UPLC^®^ HSS T3 column (100 mm × 2.1 mm I.D., particle size dp: 1.8 µm) in combination with an Acquity HSS T3 Vanguard pre-column (5 mm × 2.1 mm I.D., dp: 1.8 µm) both from Waters. The UPLC system was coupled to a Xevo^®^ TQ-S MS/MS system, equipped with an ESI probe operating in both the positive and negative ionization mode (all from Waters, Zellik, Belgium). Liquid chromatography and MS parameters were optimized as reported in Debevere et al. [29]. The developed UPLC-MS/MS method was in-house validated by a set of parameters that were in compliance with the recommendations set by the European Community [53] and with reference guidelines defined in other EU and FDA documents [54,55,56]. The method validation and the acceptance criteria are reported in Debevere et al. [29].

MassLynx version 4.1 software was used for data processing (Copyright^©^ 2012, Waters, Zellik, Belgium).

### 4.6. Data Modeling and Statistical Analysis

The efficacy of the mycotoxin detoxifiers at low and/or normal pH was tested in triplicate and all incubations were done in the same run. Statistical analyses were performed with RStudio version 1.2.5001 (Copyright^©^ 2009–2018 R studio Inc., Boston, MA, USA) using R version 3.6.1. The mycotoxin disappearance was assessed by a multilevel analysis using step forward model building including random intercept of replicate, time and time² as first level, pH, treatment, and their interaction as second level, random slope of time and two-way interactions between time and second level effects. All lower level interactions were included in the model if a higher-level interaction was significant. Random factors, i.e., replicates and time, had no/limited effect on the model. All analyses were performed using the lmer function in the lme4 package version 1.1–17 [57]. The model fit was evaluated by the Akaike information criterion (AIC). For each model, R²_m_ (marginal R², variance explained by fixed factors) was calculated using the MuMIn-package version 1.43.6 [58]. Model estimators are represented with standard error (SE) in the tables. Values of *p* < 0.05 were considered statistically significant. Graphs were constructed using ggplot2 package version 2.2.1 [59].

## Figures and Tables

**Table 1:** Comparison of the effect of three binders on the disappearance of the mycotoxins enniatin B (ENN B), roquefortine C (ROQ-C), mycophenolic acid (MPA), deoxynivalenol (DON), nivalenol (NIV), and zearalenone (ZEN) and the metabolite deepoxy-deoxynivalenol (DOM-1) during an in vitro rumen simulation study of 48 h. Fields marked with a plus-sign (+) mean that the binder adsorbs the mycotoxin to a certain extent, fields mentioning “No” mean that no statistical/relevant effect was seen in the presence of the binder.

Mycotoxin	Binder 1	Binder 2	Binder 3
ENN B	+	+	+
ROQ-C	+	No	No
MPA	No	No	No
DON	No	No	No
DOM-1	No	No	No
NIV	No	No	No
ZEN	No	No	No

**Table 2:** Ingredients (expressed in kg) of the ration given to rumen fluid donor cows used to test mycotoxin binders and modifiers in an in vitro rumen model. Study 1: binders added to feed with normal rumen pH (6.8). Study 2: ZenA added to feed with normal rumen pH (6.8). Study 3: ZenA added to rumen fluid with normal (6.8) and low (5.8) rumen pH. Study 4: BBSH 797 added to rumen fluid with normal (6.8) and low (5.8) rumen pH. Indication of “/” means that the component was not present in the diet.

Diet Components (kg)	Study 1 (Lactating Cow *n*°)			Study 2 (Lactating Cow *n*°)			Study 3 & 4 (Dry Cow *n*°)	
	*n*° 1	*n*° 2	*n*° 3	*n*° 1	*n*° 2	*n*° 3	*n*° 1	*n*° 2
Maize silage	22.62	22.62	21.3	22.62	21.3	21.3	17.3	17.3
Grass silage	18.06	18.06	14.5	18.06	14.5	14.5	20.1	20.1
Maize meal	0.85	0.85	2.0	0.85	2.0	2.0	0.9	0.9
Sugar beet pulp	/	/	8.7	/	8.7	8.7	7.2	7.2
Straw	/	/	0.3	/	0.3	0.3	0.2	0.2
Soybean meal	/	/	1.4	/	1.4	1.4	1.7	1.7
Crushed barley	/	/	0.2	/	0.2	0.2	0.24	0.24
Sodium bicarbonate	/	/	0.2	/	0.2	0.2	0.2	0.2

## Supplementary Materials

The following are available online at https://www.mdpi.com/2072-6651/12/6/405/s1, Figure S1: Effect of three different binders on the enniatin B (ENN B) concentration in the in vitro rumen model during an incubation period of 48 h, Figure S2: Effect of three different binders on the roquefortine C (ROQ-C) concentration in the in vitro rumen model during an incubation period of 48 h, Figure S3: Effect of three different binders on the mycophenolic acid (MPA) concentration in the in vitro rumen model during an incubation period of 48 h, Figure S4: Effect of three different binders on the deoxynivalenol (DON) concentration in the in vitro rumen model during an incubation period of 48 h, Figure S5: Effect of three different binders on the molar deepoxy-deoxynivalenol (DOM-1) concentration in the in vitro rumen model during an incubation period of 48 h, Figure S6: Effect of three different binders on the nivalenol (NIV) concentration in the in vitro rumen model during an incubation period of 48 h, Figure S7: Effect of three different binders on the zearalenone (ZEN) concentration in the in vitro rumen model during an incubation period of 48 h, Figure S8: Effect of three different binders on the molar α-zearalenol (α-ZEL) concentration in the in vitro rumen model during an incubation period of 48 h, Table S1: Overview of parameter estimates of the model to determine the ENN B concentration expressed relative to the maximal ENN B concentration during an in vitro rumen simulation study, Table S2: Overview of parameter estimates of the model to determine the ROQ-C concentration expressed relative to the maximal ROQ-C concentration during an in vitro rumen simulation study, Table S3: Overview of parameter estimates of the model to determine the MPA concentration expressed relative to the maximal MPA concentration during an in vitro rumen simulation study, Table S4: Overview of parameter estimates of the model to determine the DON and DOM-1 concentration expressed relative to the maximal DON concentration during an in vitro rumen simulation study, Table S5: Overview of parameter estimates of the model to determine the NIV concentration expressed relative to the maximal NIV concentration during an in vitro rumen simulation study, Table S6: Overview of parameter estimates of the model to determine the ZEN and α-ZEL concentration expressed relative to the maximal ZEN concentration during an in vitro rumen simulation study, Table S7: Overview of parameter estimates of the model to determine the ZEN and α-ZEL concentration expressed relative to the maximal ZEN concentration during an in vitro rumen simulation study, Table S8: Overview of parameter estimates of the model to determine the ZEN concentration expressed relative to the maximal ZEN concentration during an in vitro rumen simulation study, Table S9: Overview of parameter estimates of the model to determine the molar α-ZEL and β-ZEL concentration expressed relative to the maximal molar ZEN concentration during an in vitro rumen simulation study, Table S10: Overview of parameter estimates of the model to determine the molar DON and DOM-1 concentration expressed relative to the maximal molar DON concentration during an in vitro rumen simulation study, Table S11: Overview of parameter estimates of the model to determine the NIV concentration expressed relative to the maximal NIV concentration during an in vitro rumen simulation study.

## Abbreviations

α-ZAL	α-zearalanol
α-ZEL	α-zearalenol
β-ZAL	β-zearalanol
β-ZEL	β-zearalenol
DHZEN	decarboxylated hydrolyzed zearalenone
DOM-1	deepoxy-deoxynivalenol
DON	deoxynivalenol
ENN B	enniatin B
HZEN	hydrolyzed zearalenone
MPA	mycophenolic acid
NIV	nivalenol
ROQ-C	roquefortine C
VFA	volatile fatty acids
ZAN	zearalanone
ZEN	zearalenone
