# Supplementary material for: Evaluation of the Efficacy of Mycotoxin Modifiers and Mycotoxin Binders by Using an In Vitro Rumen Model as a First Screening Tool
Source: Toxins (Basel). 2020 Jun 19;12(6):405. doi: 10.3390/toxins12060405 (PMC7354577; doi:10.3390/toxins12060405)
Supplement: Supplementary file 1 [file toxins-12-00405-s001.pdf]

Supplementary Materials: Evaluation of the Efficacy of Mycotoxin Modifiers and Mycotoxin Binders by Using an In Vitro Rumen Model as a First Screening Tool

Sandra Debevere, Dian Schatzmayr, Nicole Reisinger, Markus Aleschko, Geert Haesaert, Michael Rychlik, Siska Croubels and Veerle Fievez

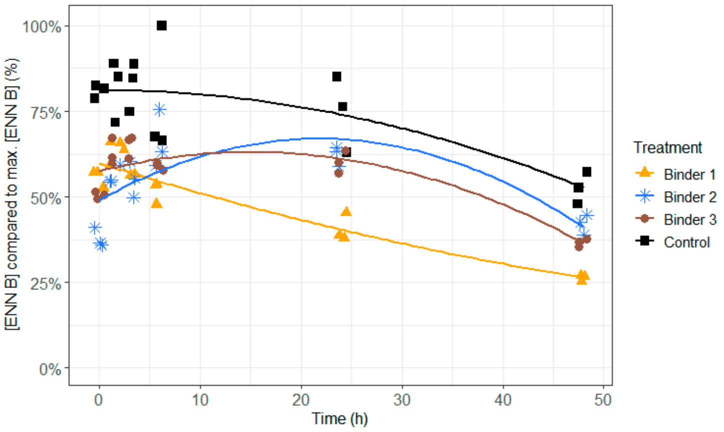

Figure S1. Effect of three different binders on the enniatin B (ENN B) concentration in the in vitro rumen model during an incubation period of 48 hours. The ENN B concentration is expressed relative to the maximum concentration detected.

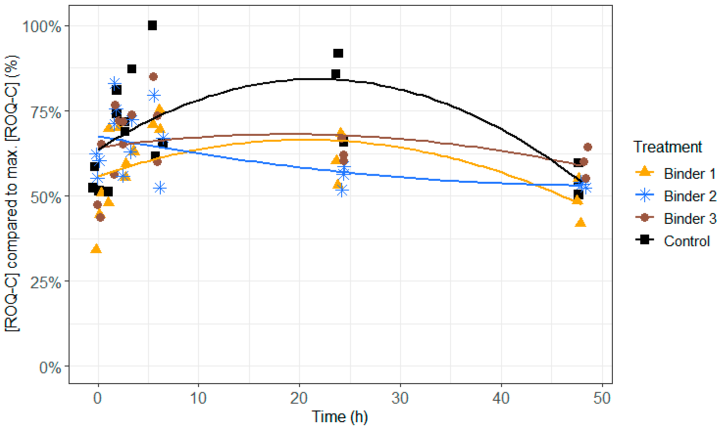

Figure S2. Effect of three different binders on the roquefortine C (ROQ-C) concentration in the in vitro rumen model during an incubation period of 48 hours. The ROQ-C concentration is expressed relative to the maximum concentration detected.

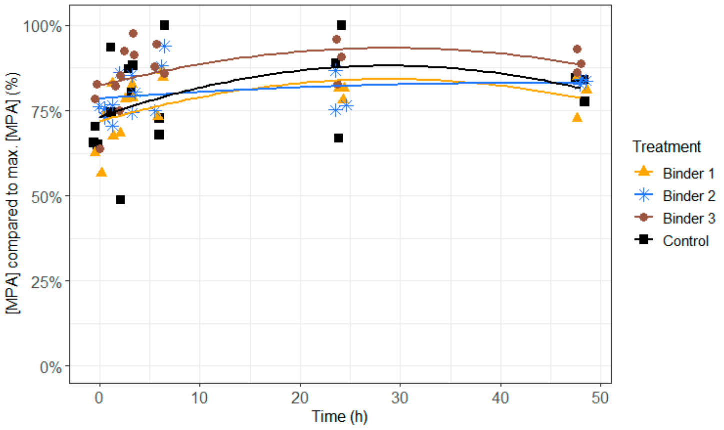

Figure S3. Effect of three different binders on the mycophenolic acid (MPA) concentration in the in vitro rumen model during an incubation period of 48 hours. The MPA concentration is expressed relative to the maximum concentration detected.

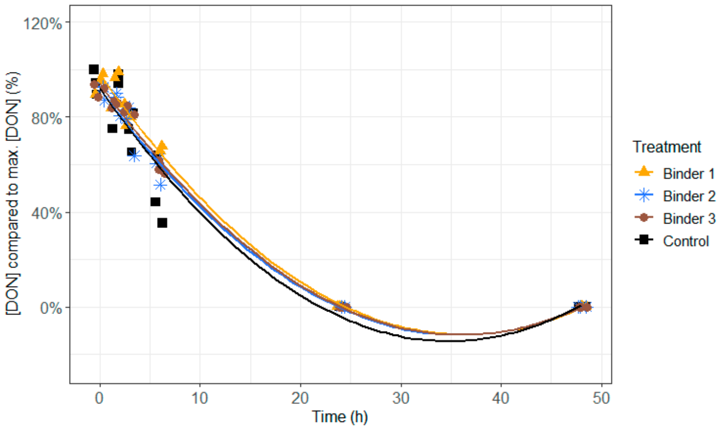

Figure S4. Effect of three different binders on the deoxynivalenol (DON) concentration in the in vitro rumen model during an incubation period of 48 hours. The DON concentration is expressed relative to the maximum concentration detected. Negative values produced by the model are obviously irrelevant and correspond with 0%.

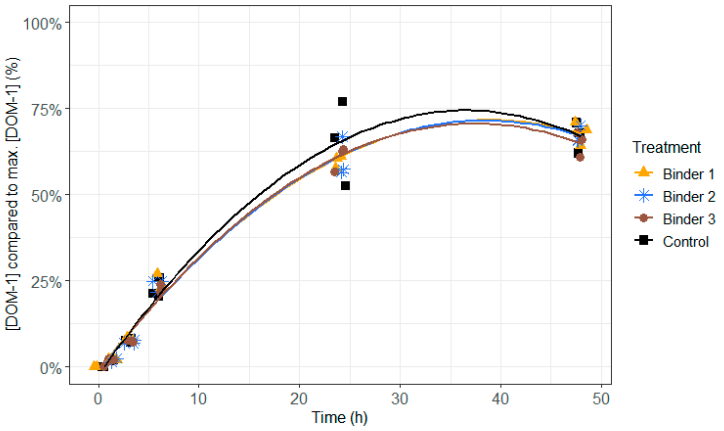

Figure S5. Effect of three different binders on the molar deepoxy-deoxynivalenol (DOM-1) concentration in the in vitro rumen model during an incubation period of 48 hours. The DOM-1 molar concentration is expressed relative to the maximum molar DON concentration detected.

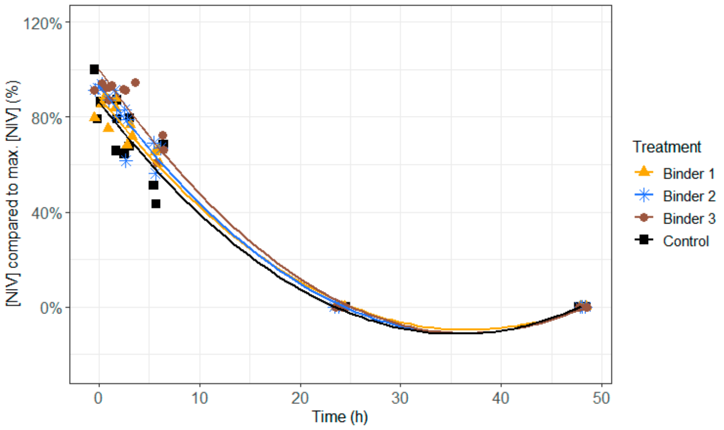

Figure S6. Effect of three different binders on the nivalenol (NIV) concentration in the in vitro rumen model during an incubation period of 48 hours. The NIV concentration is expressed relative to the maximum NIV concentration detected. Negative values produced by the model are obviously irrelevant and correspond with 0%.

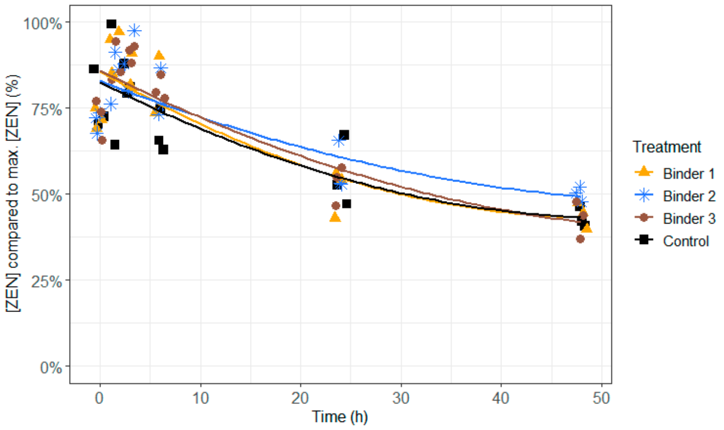

Figure S7. Effect of three different binders on the zearalenone (ZEN) concentration in the in vitro rumen model during an incubation period of 48 hours. The ZEN concentration is expressed relative to the maximum ZEN concentration detected.

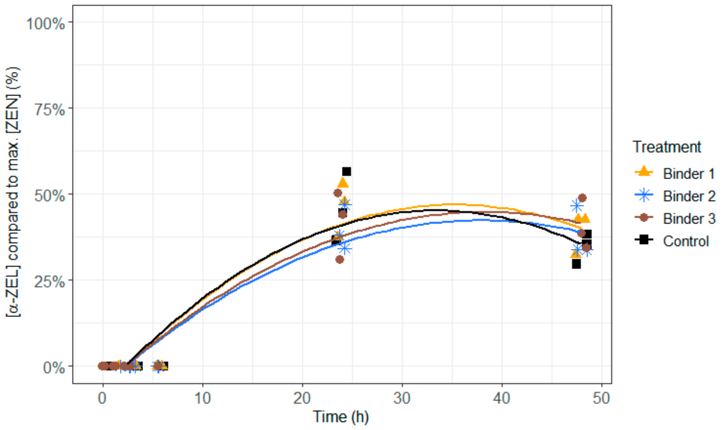

Figure S8. Effect of three different binders on the molar α-zearalenol (α-ZEL) concentration in the in vitro rumen model during an incubation period of 48 hours. The molar α-ZEL concentration is expressed relative to the maximum molar ZEN concentration detected.

Table S1. Overview of parameter estimates of the model to determine the ENN B concentration expressed relative to the maximal ENN B concentration during an in vitro rumen simulation study. In this study, the effect of three different binders on disappearance of ENN B was investigated during an incubation period of 48 hours. $R^2_m = 0.786$.

Fixed effect	Estimate	SE	P-value
Intercept	0.803	0.0227	<0.001
Time	2.43×10^{-3}	2.229×10^{-3}	0.275
Time²	-1.75×10^{-4}	4.250×10^{-5}	<0.001
Binder 1	−0.240	0.0307	<0.001
Binder 2	−0.281	0.0307	<0.001
Binder 3	−0.217	0.0307	<0.001
Time × Binder 1	-1.19×10^{-3}	1.388×10^{-3}	0.390
Time × Binder 2	4.51×10^{-3}	1.388×10^{-3}	0.00116
Time × Binder 3	1.65×10^{-3}	1.388×10^{-3}	0.235

Table S2. Overview of parameter estimates of the model to determine the ROQ-C concentration expressed relative to the maximal ROQ-C concentration during an in vitro rumen simulation study. In this study, the effect of three different binders on disappearance of ROQ-C was investigated during an incubation period of 48 hours. $R^2_m = 0.256$.

Fixed effect	Estimate	SE	P-value
Intercept	0.679	0.0330	<0.001
Time	6.98×10^{-3}	2.898×10^{-3}	0.0160
Time²	-1.86×10^{-4}	5.98×10^{-5}	0.00183
Binder 1	−0.107	0.0338	0.00155
Binder 2	−0.589	0.0338	0.0809
Binder 3	-3.87×10^{-2}	3.375×10^{-2}	0.252

Table S3. Overview of parameter estimates of the model to determine the MPA concentration expressed relative to the maximal MPA concentration during an in vitro rumen simulation study. In this study, the effect of three different binders on disappearance of MPA was investigated during an incubation period of 48 hours. $R^2_m = 0.264$.

Fixed effect	Estimate	SE	P-value
Intercept	0.747	0.0232	<0.001
Time	7.14×10^{-3}	2.442×10^{-3}	0.00350
Time²	-1.21×10^{-4}	5.05×10^{-5}	0.0167
Binder 1	-2.25×10^{-2}	2.849×10^{-2}	0.430
Binder 2	1.60×10^{-2}	2.849×10^{-2}	0.575
Binder 3	7.74×10^{-2}	2.849×10^{-2}	0.00656

Table S4. Overview of parameter estimates of the model to determine the DON and DOM-1 concentration expressed relative to the maximal DON concentration during an in vitro rumen simulation study. In this study, the effect of three different binders on disappearance of DON and the metabolite DOM-1, formed out of the parent molecule DON, was investigated during an incubation period of 48 hours. $R^2_m = 0.978$ for DON and 0.984 for DOM-1.

Fixed effect	DON			DOM-1		
	Estimate	SE	P-value	Estimate	SE	P-value
Intercept	0.922	0.0158	<0.001	-1.00×10^{-2}	9.65×10^{-3}	0.298
Time	-5.97×10^{-2}	1.67×10^{-3}	<0.001	3.89×10^{-2}	1.01×10^{-3}	<0,001
Time²	8.35×10^{-4}	3.44×10^{-5}	<0.001	-5.15×10^{-4}	2.08×10^{-5}	<0,001
Binder 1	4.86×10^{-2}	1.943×10^{-2}	0.0124	-3.90×10^{-3}	1.1734×10^{-2}	0.739
Binder 2	9.60×10^{-3}	1.9429×10^{-2}	0.621	-6.59×10^{-3}	1.1734×10^{-2}	0.574
Binder 3	2.19×10^{-2}	1.943×10^{-2}	0.259	-1.08×10^{-2}	1.173×10^{-2}	0.356

Table S5. Overview of parameter estimates of the model to determine the NIV concentration expressed relative to the maximal NIV concentration during an in vitro rumen simulation study. In this study, the effect of three different binders on disappearance of NIV was investigated during an incubation period of 48 hours. $R^2_m = 0.978$.

Fixed effect	Estimate	SE	P-value
Intercept	8.66×10^{-1}	1.82×10^{-2}	<0.001
Time	-5.54×10^{-2}	1.76×10^{-3}	<0.001
Time²	7.80×10^{-4}	3.36×10^{-5}	<0.001
Binder 1	2.78×10^{-2}	2.421×10^{-2}	0.251
Binder 2	6.67×10^{-2}	2.421×10^{-2}	0.00587
Binder 3	1.28×10^{-1}	2.421×10^{-2}	<0.001
Time × Binder 1	-5.47×10^{-4}	1.0959×10^{-3}	0.617
Time × Binder 2	-1.59×10^{-3}	1.096×10^{-3}	0.147
Time × Binder 3	-3.08×10^{-3}	1.096×10^{-3}	0.00500

Table S6. Overview of parameter estimates of the model to determine the ZEN and α-ZEL concentration expressed relative to the maximal ZEN concentration during an in vitro rumen simulation study. In this study, the effect of three different binders on disappearance of ZEN and the metabolite α-ZEL, formed out of the parent molecule ZEN, was investigated during an incubation period of 48 hours. R^2_m = 0.732 for ZEN and 0.897 for α-ZEL.

	ZEN			α-ZEL		
**Fixed effect**	**Estimate**	**SE**	**P-value**	**Estimate**	**SE**	**P-value**
Intercept	0.826	0.0246	<0,001	−0.0628	0.01721	<0,001
Time	-1.45×10^{-2}	2.61×10^{-3}	<0,001	2.82×10^{-2}	1.82×10^{-3}	<0,001
Time²	1.29×10^{-4}	5.38×10^{-5}	0.0163	-3.92×10^{-4}	3.75×10^{-5}	<0,002
Binder 1	1.74×10^{-2}	3.080×10^{-2}	0.572	8.15×10^{-3}	2.1199×10^{-2}	0.700
Binder 2	2.82×10^{-2}	3.036×10^{-2}	0.353	-3.92×10^{-3}	2.1199×10^{-2}	0.853
Binder 3	2.43×10^{-2}	3.036×10^{-2}	0.424	3.75×10^{-3}	2.1199×10^{-2}	0.860

Table S7. Overview of parameter estimates of the model to determine the ZEN and α-ZEL concentration expressed relative to the maximal ZEN concentration during an in vitro rumen simulation study. In this study, the effect of a mycotoxin detoxifying enzyme added to the feed on the disappearance of ZEN and the formation of the metabolite α-ZEL out of the parent molecule ZEN was investigated at two different concentrations (125 µg/mg feed and 62.5 µg/mg feed) during an incubation period of 48 hours. R^2_m = 0.975 for ZEN and 0.854 for α-ZEL.

Fixed effect	ZEN			α ZEL		
	**Estimate**	**SE**	**P-value**	**Estimate**	**SE**	**P-value**
Intercept	0.830	0.0176	<0.001	−0.0245	0.02066	0.236
Time	-1.31×10^{-2}	1.92×10^{-3}	<0.001	2.08×10^{-2}	2.25×10^{-3}	<0.001
Time²	8.87×10^{-5}	3.755×10^{-5}	0.0181	-1.76×10^{-4}	4.40×10^{-5}	<0.001
High conc.	−0.816	0.0235	<0.001	-3.59×10^{-3}	2.7479×10^{-2}	0.896
Low conc.	−0.816	0.0235	<0.001	-3.59×10^{-3}	2.7479×10^{-2}	0.896
Time × High conc.	8.94×10^{-3}	1.062×10^{-3}	<0.001	-1.25×10^{-2}	1.24×10^{-3}	<0.001
Time × Low conc.	8.94×10^{-3}	1.062×10^{-3}	<0.001	-1.25×10^{-2}	1.24×10^{-3}	<0.001

Table S8. Overview of parameter estimates of the model to determine the ZEN concentration expressed relative to the maximal ZEN concentration during an in vitro rumen simulation study. In this study, the effect of a mycotoxin detoxifying enzyme added to a rumen fluid-buffer mixture with low (5.8) or normal (6.8) pH on the disappearance of ZEN was investigated at two different concentrations (125 µg/mg feed and 62.5 µg/mg feed) during an incubation period of 48 hours. R^2_m = 0.976.

Fixed effect	Estimate	SE	P-value
Intercept	0.985	0.0883	<0.001
Time	−0.0439	0.01117	<0.001
Time²	5.41×10^{-3}	1.727×10^{-3}	0.002
High conc.	−0.790	0.0169	<0.001
Low conc.	−0.776	0.0169	<0.001
Buffer	−0.0187	0.01378	0.175

Table S9. Overview of parameter estimates of the model to determine the molar α-ZEL and β-ZEL concentration expressed relative to the maximal molar ZEN concentration during an in vitro rumen simulation study. In this study, the effect of a mycotoxin detoxifying enzyme added to a rumen fluid-buffer mixture with low (5.8) or normal (6.8) pH on the formation of the metabolites α-ZEL and β-ZEL out of the parent molecule ZEN was investigated at two different concentrations (125 μg/mg feed and 62.5 μg/mg feed) during an incubation period of 48 hours. R^2_m = 0.883 for α-ZEL and 0.767 for β-ZEL.

**Fixed effect**	**α-ZEL**			**β-ZEL**		
	**Estimate**	**SE**	**P-value**	**Estimate**	**SE**	**P-value**
Intercept	−0.0319	0.04880	0.514	−4.39 × 10⁻³	3.2134 × 10⁻²	0.891
Time	−0.138	0.0142	<0.001	−6.23 × 10⁻²	9.35 × 10⁻³	<0.001
High conc.	0.0319	0.0690	0.644	4.39 × 10⁻³	4.5439 × 10⁻²	0.923
Low conc.	0.0319	0.0690	0.644	4.39 × 10⁻³	4.54390 × 10⁻²	0.923
Buffer	5.49 × 10⁻³	7.722 × 10⁻³	0.477	7.57 × 10⁻⁴	5.0841 × 10⁻³	0.882
High conc. × buffer	−5.49 × 10⁻³	1.0921 × 10⁻²	0.615	−7.57 × 10⁻⁴	7.1899 × 10⁻³	0.916
Low conc. × buffer	−5.49 × 10⁻³	1.0921 × 10⁻²	0.615	−7.57 × 10⁻⁴	7.1899 × 10⁻³	0.916
High conc. × time	0.138	0.0201	<0.001	6.23 × 10⁻²	1.322 × 10⁻²	<0.001
Low conc. × time	0.138	0.0201	<0.001	6.23 × 10⁻²	1.322 × 10⁻²	<0.001
Buffer × time	0.0238	0.00225	<0.001	1.07 × 10⁻²	1.48 × 10⁻³	<0.001
High conc. × buffer × time	−0.0238	0.00318	<0.001	−1.07 × 10⁻²	2.09 × 10⁻³	<0.001
Low conc. × buffer × time	−0.0238	0.00318	<0.001	−1.07 × 10⁻²	2.09 × 10⁻³	<0.001

Table S10. Overview of parameter estimates of the model to determine the molar DON and DOM-1 concentration expressed relative to the maximal molar DON concentration during an in vitro rumen simulation study. In this study, the effect of a mycotoxin detoxifying bacterial strain BBSH 797 added to a rumen fluid-buffer mixture with low (5.8) or normal (6.8) pH on the DON disappearance and DOM-1 production out of the parent molecule DON was investigated during an incubation period of 48 hours. R^2_m = 0.954 for DON and 0.982 for DOM-1.

**Fixed effect**	**DON**			**DOM-1**		
	**Estimate**	**SE**	**P-value**	**Estimate**	**SE**	**P-value**
Intercept	0.926	0.1445	<0.001	−2.72 × 10⁻²	7.576 × 10⁻²	0.719
Time	8.23 × 10⁻²	6.692 × 10⁻³	<0.001	−7.34 × 10⁻²	3.51 × 10⁻³	<0.001
Time²	1.58 × 10⁻⁴	3.13 × 10⁻⁵	<0.001	−1.03 × 10⁻⁴	1.64 × 10⁻⁵	<0.001
BBSH present	3.37 × 10⁻¹	2.240 × 10⁻¹	0.132	−0.117	0.1174	0.319
Buffer	−1.66 × 10⁻²	2.286 × 10⁻²	0.469	3.08 × 10⁻³	1.1984 × 10⁻²	0.797
BBSH × buffer	−6.10 × 10⁻²	3.544 × 10⁻²	0.0851	2.09 × 10⁻²	1.858 × 10⁻²	0.260

BBSH × time	−3.01 × 10^{-2}	9.63 × 10^{-3}	0.00175	2.10 × 10^{-2}	5.05 × 10^{-3}	<0.001
Buffer × time	−1.55 × 10^{-2}	1.03 × 10^{-3}	<0.001	1.36 × 10^{-2}	5.4 × 10^{-4}	<0.001
BBSH × buffer × time	4.30 × 10^{-3}	1.524 × 10^{-3}	0.00483	−3.03 × 10^{-3}	7.99 × 10^{-4}	<0.001

Table S11. Overview of parameter estimates of the model to determine the NIV concentration expressed relative to the maximal NIV concentration during an in vitro rumen simulation study. In this study, the effect of a mycotoxin detoxifying bacterial strain BBSH 797 added to a rumen fluid-buffer mixture with low (5.8) or normal (6.8) pH on the NIV production was investigated during an incubation period of 48 hours. R^2_m = 0.789.

**Fixed effect**	**Estimate**	**SE**	**P-value**
Intercept	0.899	0.2535	<0.001
Time	1.76 × 10^{-2}	1.174 × 10^{-2}	0.133
Time^2	3.10 × 10^{-4}	5.49 × 10^{-5}	<0.001
BBSH present	0.439	0.3929	0.264
Buffer	−5.69 × 10^{-2}	4.010 × 10^{-2}	0.156
BBSH × buffer	−7.57 × 10^{-2}	6.216 × 10^{-2}	0.223
BBSH × time	−3.47 × 10^{-2}	1.690 × 10^{-2}	0.0401
Buffer × time	−6.12 × 10^{-3}	1.815 × 10^{-3}	<0.001
BBSH × buffer × time	5.04 × 10^{-3}	2.674 × 10^{-3}	0.0593
